# Editorial: Thrombosis meets inflammation

**DOI:** 10.3389/fimmu.2023.1303385

**Published:** 2023-10-18

**Authors:** Krasimir Kolev, Robert L. Medcalf

**Affiliations:** ^1^ Department of Biochemistry, Semmelweis University, Budapest, Hungary; ^2^ Australian Centre for Blood Diseases, Monash University, Melbourne, VIC, Australia

**Keywords:** hemostasis, fibrin, complement system, COVID-19, staphylocoagulase, neutrophil extracellular traps, air embolism

The hemostatic and immune systems are tightly coupled in the fight against pathogens (1). From an evolutionary perspective, lower-level invertebrates use a single cell type (“nucleated immunohemostatic cells”) to protect themselves from pathogens and to prevent the leakage of hemolymphs from the vascular bed. In higher-order organisms, these two functions diverge into more specialized systems: blood coagulation factors, platelets, endothelium at the hemostatic end, the complement system, leukocytes, and antibodies at the immunity side. The coordinated action of these systems is preserved through a complex crosstalk of multiple molecular and cellular interactions. However, in many medical conditions, this intricately balanced interplay can be disturbed, for example, by overshooting systemic or local inflammation that can result in undesired intravascular blood clot formation (a process referred to as “thromboinflammation”). In recent years, coronavirus disease 2019 (COVID-19)–associated coagulopathy, the most devastating complication of the SARS-CoV-2 infection, has incited intensive research in the field, which confirmed the pathogenic role of neutrophil extracellular traps (NETs) at the interface of thrombosis and inflammation. The prothrombotic role of NETs had already emerged from earlier studies on diseases posing the highest public health burden worldwide (cardio- and cerebrovascular diseases and deep vein thrombosis) (2). This Research Topic presents a collection of articles that report new original findings or review previous research, providing new insights into the relationship between inflammation and thrombosis.

Marsh et al. present an in-depth and nicely illustrated review of thromboinflammatory events in iatrogenic vascular air embolism, a complication with significant mortality in many clinical settings (neurosurgery, cardiac surgery, liver transplantation, endoscopy, hemodialysis, thoracentesis, tissue biopsy, and angiography). In these cases, air bubbles arrive on the arterial side of the circulation and lodge in an end organ, most lethally in the cerebral or coronary circulation. There, the air bubbles adhere to and interact with endothelial glycocalyx components (hyaluronan and heparan sulfate) and air-mediated complement C3 activation is associated with the release of proinflammatory cytokines and tissue factor. The changed fluid dynamics and increased shear rate contribute to platelet activation around the emboli. Thus, this review clearly delineates the rationale for anticoagulation, antiplatelet, and anticomplement therapy in air embolism.

With elegant *in vitro* experiments, Burmeister et al. characterize a self-accelerating circuit of the effects of extended DNA fiber networks on blood flow, thrombus formation, and complement activation. This study demonstrates that local activation of the complement system at the vascular wall triggers NETs formation. The released DNA strands decelerate blood flow and induce blood clots independent of plasmatic coagulation but accompanied by complement activation.

Because NETs are known to provoke thrombosis, their removal from the circulation is crucial to prevent uncontrolled thromboinflammation. To improve the clearance of the major NET component, Englert et al. engineer a dual-active DNase, combining DNase1 and DNase1L3 activities. These authors demonstrate that the recombinant enzyme is superior to DNase1 and DNase1L3 alone in degrading human NETs. Importantly, the *in vivo* relevance of the dual-active DNase is supported by its stability in circulation following its transgenic expression in DNase-deficient mice.

Komorowicz et al. approach thromboinflammation from the pathogen’s perspective. Because the concept of thromboinflammation suggests that both fibrin and NETs might be important in immobilizing, and perhaps eliminating bacteria, this study reveals the molecular mechanisms through which bacteria interfere with the NET-related modulation of blood clotting and thrombus-resolution. *Staphylococcus aureus* is known to contribute to NET formation via its virulence factors and, in parallel, through its staphylocoagulase that generates a fibrin variant (SCG-fibrin) with a structure different from that of the endogenous fibrin. This study demonstrates that the major NET components (DNA-histone complexes) reduce the mechanical stability of SCG-fibrin and probably compromise its function as a mechanical barrier. Thus, the NET machinery strikes back at the host and helps *Staphylococci* to evade the immune defense. In addition, the DNA-histone complexes render the SCG-fibrin more resistant to fibrinolysis, prolonging the lifespan of this pro-pathogenic environment.

The article by Fields et al. looks at a different picture, and provides some new insights into the role of platelet releasates in the promotion of a thromboinflammatory phenotype in COVID-19. Here, the authors demonstrate that the addition of plasma from patients with COVID-19 to platelets or neutrophils from normal individuals alters platelet and neutrophil behavior; for the latter, this includes the promotion of NETs. The factor(s) responsible for promoting these effects remains to be identified, although PAI-1 is considered as a possible candidate soluble factor.

Jing et al. provide a review highlighting the possible mechanisms of long COVID thromboembolic complications, the role of the inflammatory response, and the therapeutic strategies. Much discussion is raised about optimal anti-platelet and anticoagulation treatments.

Laat-Kremers et al. also provide a fascinating perspective on coagulation factors and other indices that might be predictive of COVID-19-related thrombosis. Here, the authors develop a neural network that incorporates various indices, including, among others, C-reactive protein, sex, age, thrombin generation, α_2_-macroglobulin, IgM, plasmin generation, and thrombin-antithrombin complexes. The neural network is created using artificial intelligence and generates a predictive value of thrombosis in patients with COVID-19 that, remarkably, approaches almost 100%.

Finally, Bagoly et al. present an interesting study on the effect of the anti-SARS-CoV-2 BNT162b2 mRNA vaccine on thrombin generation in a pediatric population with inflammatory bowel disease (IBD), a condition that increases the risk of thrombosis. This study demonstrates that vaccination is safe and efficacious in children with IBD. Indeed, thrombin generation parameters, inflammatory markers, and IBD severity score do not increase significantly after mRNA vaccination. This study therefore supports the safety of SARS-CoV-2 mRNA vaccination in children with IBD.

We look forward to future research into this ever-evolving field of thromboinflammation.

## Author contributions

KK: Writing – original draft, Writing – review & editing. RM: Writing – original draft, Writing – review & editing.
